# Selenium and Glutathione-Depleted Rats as a Sensitive Animal Model to Predict Drug-Induced Liver Injury in Humans

**DOI:** 10.3390/ijms20133141

**Published:** 2019-06-27

**Authors:** Keisuke Goda, Kyotaka Muta, Yuzo Yasui, Shin-ichi Oshida, Kanae Kitatani, Susumu Takekoshi

**Affiliations:** 1 Toxicology Research Lab., Central Pharmaceutical Research Institute, JAPAN TOBACCO INC., 1-13-2 Fukuura, Kanazawa, Yokohama, Kanagawa 236-0004, Japan; 2 Department of Cell Biology, Division of Host Defense Mechanism, Tokai University School of Medicine, 143 Shimokasuya, Isehara, Kanagawa 259-1193, Japan

**Keywords:** drug-induced liver injury, selenium, glutathione, animal model, oxidative stress

## Abstract

Drug-induced liver injury (DILI) is one of the most serious and frequent drug-related adverse events in humans. Selenium (Se) and glutathione (GSH) have a crucial role for the hepatoprotective effect against reactive metabolites or oxidative damage leading to DILI. The hepatoprotective capacity related to Se and GSH in rodents is considered to be superior compared to the capacity in humans. Therefore, we hypothesize that Se/GSH-depleted rats could be a sensitive animal model to predict DILI in humans. In this study, Se-deficiency is induced by feeding a Se-deficient diet and GSH-deficiency is induced by l-buthionine-*S,R*-sulfoxinine treatment via drinking water. The usefulness of this animal model is validated using flutamide, which is known to cause DILI in humans but not in intact rats. In the Se/GSH-depleted rats from the present study, decreases in glutathione peroxidase-1 protein expression and GSH levels and an increase in malondialdehyde levels in the liver are observed without any increase in plasma liver function parameters. Five-day repeated dosing of flutamide at 150 mg/kg causes hepatotoxicity in the Se/GSH-depleted rats but not in normal rats. In conclusion, Se/GSH-depleted rats are the most sensitive for detecting flutamide-induced hepatotoxicity in all the reported animal models.

## 1. Introduction

Drug-induced liver injury (DILI) is one of the most serious and frequent drug-related adverse events in non-clinical toxicity studies or in clinical settings and a main reason for regulatory action pertaining to drugs, including stopping clinical trials or withdrawal from the marketplace [1,2]. Therefore, an estimation of the potential risk of drug candidates to induce DILI as a result of non-clinical toxicity study is important to facilitate the development of new drugs. However, the prediction of the risk of DILI in humans is difficult from the results of non-clinical toxicity studies using normal animals, especially rodents.

Selenium (Se) is an essential trace element in mammals. The functional role of Se in biological processes is largely attributable to its incorporation into selenoproteins [3]. Glutathione peroxidase (GPx), which is one of the most important and typical selenoproteins, plays a crucial role in the detoxification of reactive metabolites and the prevention of cellular oxidative damage [4]. Eight GPx isoforms have been identified in mammals. GPx-1 is ubiquitously expressed in almost all tissues and is a major subtype of GPx isoforms [5,6]. GPx-1 is localized in cytosol and mitochondria and plays a significant role in the degeneration of hydrogen peroxide (H_2_O_2_) and fatty acid hydroperoxides, especially in mitochondria because mitochondria lack catalase, which is another enzyme degenerating H_2_O_2_ [7,8,9]. In addition, peroxynitrite, which is a peroxide of nitrogen monoxide (NO), is a strong inducer of oxidative stress and inflammatory reactions [10]. GPx is known to have not only preventative effects on oxidative damage induced by peroxynitrite, but also reductive effects on cellular peroxynitrite levels [11]. GPx1-deficient mice are known to be more susceptible to peroxynitrite-induced apoptosis of hepatocytes than wild-type mice [12]. Hepatic GPx levels are known to decrease markedly following the feeding of Se-deficient diet to rats [13].

Glutathione (GSH) is a tripeptide of cysteine, glycine, and glutamic acid and has a potent anti-oxidative capacity. GSH plays an important role in not only protecting tissues against the degenerative effects of oxidative damage by scavenging free radicals from endogenous or exogenous compounds [14], but also by directly detoxifying many exogenous compounds, such as acetaminophen (APAP) and methimazol [15,16]. For instance, APAP is metabolized by cytochrome P450 (CYP) to generate a highly reactive and cytotoxic intermediate N-acetyl-p-benzoquinone imine (NAPQI) when APAP is administered at high dose levels [17]. NAPQI is quickly inactivated in conjugation with GSH in high hepatic GSH level conditions. In the absence of a sufficient hepatic GSH level because of malnutrition, exposure to high oxidative stress or genetic background, reactive NAPQI can cause DILI [18,19,20]. l-buthionine-*S,R*-sulfoxinine (BSO), a well-known irreversible inhibitor of γ-glutamylcysteine synthetase (γ-GCS), markedly decreases hepatic GSH levels without any apparent toxicity or any effects on the hepatic microsomal and cytosolic enzymes responsible for metabolism [21]. GSH-depleted animal models (rats or mice) after the treatment with BSO are well known to evaluate potential risk of DILI for several drugs including APAP, methimazole, tienilic acid, and amodiaquine that produce reactive metabolites [22,23].

Therefore, we hypothesize that Se and GSH-depleted rats could be a sensitive and novel animal model to predict DILI in humans. In this study, Se deficiency is induced by the feeding of a Se-deficient diet and GSH deficiency was induced by BSO treatment via drinking water. The usefulness of this animal model is validated using flutamide, which is known to cause DILI in humans but not in intact rats.

## 2. Results

### 2.1. Preparation of Se/GSH-Depleted Rats

**Figure 1:**
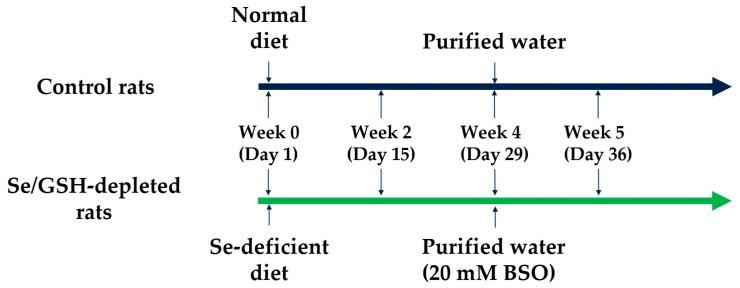
Preparing procedure of selenium/glutathione (Se/GSH)-depleted rats.

**Figure 2:**
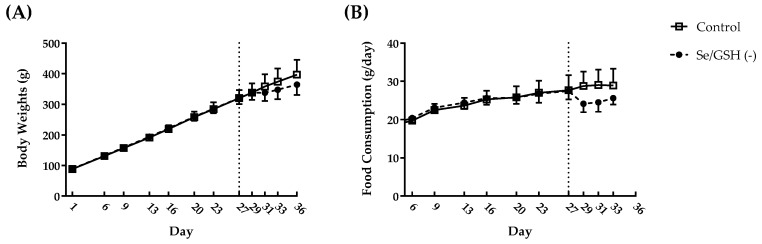
Time-course changes of the body weights (**A**) and food consumption (**B**) in the control and Se/GSH-depleted rats. (**A**) Day 1 means the day the study was initiated. (**B**) Food consumption on Day 6 was calculated between Days 1 and 6. Day 9: Days 6–9, Day 13: Days 9–13, Day 16: Days 13–16, Day 20: Days 16–20, Day 23: Days 20–23, Day 27: Days 23–27, Day 29: Days 27–29, Day 31: Days 29–31, Day 33: Days 31–33 and Day 36: Days 33–36. The dotted line for Day 27 indicates the initiation of dosing of BSO. Each point represents mean + or − S.D. with 6–12 determinations. Se/GSH (−): Se/GSH-depleted group.

Selenium and GSH-depleted rats (Se/GSH-depleted rats) were prepared according to the following procedure (Figure 1). No clinical signs were observed in the control or Se/GSH-depleted group throughout the study period. The body weights and food consumption did not change after feeding the Se deficient diet, but were slightly decreased after treatment with BSO (Figure 2).

### 2.2. Profiling of Se/GSH-Depleted Rats

**Figure 3:**
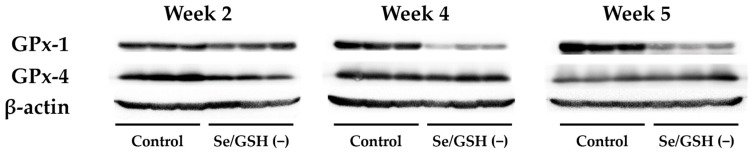
The protein expression of hepatic GPx-1 and 4 in the control and Se/GSH-depleted rats. Hepatic protein expression of GPx-1 (22 kDa), GPx-4 (22 kDa) and β-actin (45 kDa) are presented (*n* = 3/sampling points). Se/GSH (−): Se/GSH-depleted group.

Serum Se levels were significantly decreased in the Se/GSH-depleted group at all sampling points when compared with the control group (Table 1). Hepatic GPx-1 protein expression was markedly decreased in the Se/GSH-depleted group in Weeks 4 and 5 when compared with the control group, but hepatic GPx-4 protein expression was not changed by Se/GSH depletion at any sampling point (Figure 3). Hepatic GSH levels were significantly increased in the Se/GSH-depleted group in Weeks 2 and 4 when compared with the control group, but were decreased in Week 5 (Table 1). Hepatic glutathione disulfide (GSSG) levels were significantly decreased in Se/GSH-depleted group at all sampling points (Table 1). The GSH/GSSG ratio was significantly decreased in Se/GSH-depleted group in Weeks 2 and 4 when compared with the control group but did not change in Week 5 (Table 1). Hepatic malondialdehyde (MDA) levels were significantly increased in the Se/GSH-depleted group in Weeks 4 and 5 when compared with the control group (Table 1). There were no treatment-related changes in typical liver function parameters including aspartate aminotransferase (AST), alanine aminotransferase (ALT), total bilirubin (T-BIL), glutamate dehydrogenase (GLDH) and sorbitol dehydrogenase (SDH) levels in the plasma at any sampling point (Table 1).

### 2.3. Toxicity Study of Flutamide Using Se/GSH-Depleted Rats

**Figure 4:**
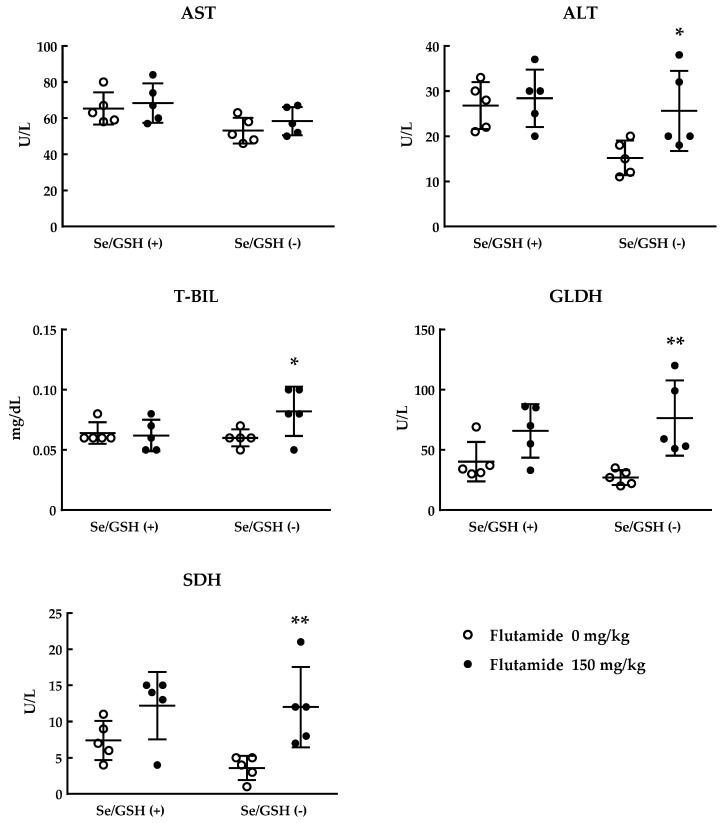
Liver function parameters in the Se/GSH (−) and (+) rats treated with flutamide. All data and mean ± S.D. are presented with 5 determinations. * *p* < 0.05, ** *p* < 0.01 (Student’s *t*-test). Se/GSH (+): normal rats, Se/GSH (−): Se/GSH-depleted rats.

The administration of flutamide for Se/GSH-depleted rats was started two days after the initiation of treatment with BSO (dosing duration of flutamide: Five days). Plasma ALT, T-BIL, GLDH and SDH levels were significantly increased in the Se/GSH-depleted (Se/GSH (−)) rats treated with flutamide when compared with the matched control group but not in intact (Se/GSH (+)) rats (Figure 4). There were no marked changes in plasma AST in the Se/GSH (−) and (+) groups treated with flutamide (Figure 4). Histopathologically, centrilobular hepatocytic hypertrophy was observed in the Se/GSH (−) and (+) rats treated with flutamide (Table 2). Only in the Se/GSH (−) group, inflammatory cell infiltration in the periportal region, single cell necrosis of centrilobular hepatocytes, and focal necrosis of hepatocytes in the subcapsular region were observed in rats treated with flutamide (Table 2). In addition, the most obvious increases in liver function parameters were observed in two animals observed with histopathological findings indicating the degeneration of their hepatocytes.

## 3. Discussion

Drug-induced liver injury (DILI) is one of the most serious and frequent drug-related adverse events in non-clinical toxicity studies or in clinical settings. DILI is classified into intrinsic and idiosyncratic types, however, intrinsic DILI is limited to a few cases in clinical practice, such as APAP-induced hepatotoxicity [24]. On the other hand, idiosyncratic DILI has become a major clinical concern because of its unpredictable nature, frequent hospitalizations, need for liver transplantation, and high mortality. The estimation of the potential for compounds to induce idiosyncratic DILI in the clinical setting is considered to be very difficult in non-clinical toxicity studies using normal animals.

One of the possible mechanisms of idiosyncratic DILI is oxidative stress induced by reactive metabolites in the liver [25]. GSH and related enzymes such as GPx play an important role in protecting the liver against the toxic effects of oxidative stress by scavenging free radicals produced from reactive metabolites [26,27]. The hepatic GSH content in rodents is about two-fold higher than in humans [28,29]. Hepatic GPx activity in rodent is also much higher than in humans [30] and serum Se level in normal rats as estimated in this study (509–607 μg/L) is about five-fold higher than the normal range in humans (107–171 μg/L). In addition, the activity of hepatic GSH S-transferase, which catalyzes GSH conjugation of reactive metabolites, is 10 to 20-fold higher in rodents than in humans [31]. Since the protective mechanism against oxidative stress leading to DILI in rodents is considered to be superior compared with that of humans based on the above-mentioned characteristics, we hypothesized that Se and GSH-depleted rats could be a sensitive and novel animal model to predict DILI in humans.

In Se/GSH-depleted rats, decreases in serum Se level and hepatic GSSG/GSH ratio were observed from Week 2. A decreased hepatic GSSG/GSH ratio was considered to indicate decreased hepatic GPx level and thereby increases in unused GSH. From Week 4, hepatic GPx-1 protein expression was markedly decreased in Se/GSH-depleted rats but hepatic GPx-4 protein expression did not change (Figure 3). GPx-1 has been known to be the most sensitive isoform for the systemic Se condition of all the GPx isoforms. Marked decreases in GPx-1 mRNA levels and protein expression are observed in low Se conditions [13]. In addition, since it has been reported that GPx-4 was not affected by the systemic Se condition [32], the results of this study were considered to be reasonable. Consistent with a decrease in hepatic GPx-1 expression, an increase in the hepatic MDA level was noted, indicating a failure of the protective mechanism against oxidative stress in the liver. The effects of Se/GSH-depletion on the animals’ physical condition were minimal based on the comparison of the body weights and food consumption for the intact rats (Figure 2). There were no increases in any liver function parameters among the Se/GSH-depleted rats (Table 1). Therefore, it was considered that the potential risk of DILI in humans could be estimated appropriately by the dosing of a test compound during BSO-treatment in Se/GSH-depleted rats.

We validated the usefulness of Se/GSH-depleted rats using flutamide. Flutamide is a widely used non-steroidal anti-androgen for the treatment of prostate cancer. Flutamide is known to cause idiosyncratic DILI in rare cases and received a black box warning label by the U.S. Food and Drug Administration [33,34,35,36]. Although many studies have been performed to elucidate the cause of the flutamide-induced hepatotoxicity, the exact mechanism remains elusive. Metabolic activation to reactive intermediates and oxidative stress are considered to partly contribute to flutamide-induced hepatotoxicity [37,38,39,40,41]. In the Se/GSH-depleted rats, plasma ALT, T-BIL, GLDH, and SDH levels were significantly increased in the Se/GSH (−) rats when compared with the matched control group but not in normal (Se/GSH (+)) rats (Figure 4). Histopathological findings indicating degeneration of the hepatocytes were observed in rats treated with flutamide only in the Se/GSH (−) group (Table 2).

Previous studies have demonstrated that dosing of flutamide at 150 mg/kg for 28 days to normal male rats did not cause hepatotoxicity [42]. Excessively high doses of flutamide (500 mg/kg for 3 days) caused only an increase in liver weight in normal rats which may reflect an adaptive response to the high dose level of the compound rather than hepatotoxicity [43]. Another study using CYP1A2 knockout mice revealed that these animals tolerated even repeated dosing of excessively high doses of flutamide (800 mg/kg for 28 days). Only in combination with severe depletion of GSH by feeding an amino acid-deficient diet to CYP1A2 knockout mice, a high dose level of flutamide (400 mg/kg for 14 days) induced a mild increase in plasma ALT level and a slight increase in single cell necrosis in the liver [44]. However, amino acid-deficiency was considered to be an extreme deviation deviated from normal physiological conditions and the utility of this model for the estimation of DILI remains controversial. Using γ-GCS knockdown rats, dosing flutamide at 100 mg/kg for seven days increased plasma AST and ALT levels, however, the increases were very slight and there were no histopathological findings suggestive of hepatotoxicity in the liver [45].

In conclusion, Se/GSH-depleted rats established in the present study are the most sensitive to the detection of the flutamide-induced hepatotoxicity in all the reported animal models.

## 4. Materials and Methods

### 4.1. Materials

BSO was purchased from Toronto Research Chemicals Inc. (North York, ON, Canada) and flutamide was purchased from Wako Pure Chemicals (Tokyo, Japan). All other reagents were obtained commercially and were the highest grade available.

### 4.2. Animals

Four-week-old male Crl:CD (SD) rats were purchased from Charles River Japan Inc. (Kanagawa, Japan). The animals were housed individually in wire-mesh cages kept in an air-conditioned room with a 12 h light-dark cycle (lighting from 7:00 a.m. to 7:00 p.m.) at a temperature of 23 ± 1 °C, a relative humidity of 55 ± 5%, and a ventilation rate of about 15 times per hour. The rats were quarantined for 1 week and were allowed free access to a commercial pelleted diet (AIN-93M, Oriental Yeast Co., Ltd., Tokyo, Japan) or Se-deficient diet (AIN-93M base, Oriental Yeast Co., Ltd.). The normal diet and Se-deficient diets used in this study contained 0.22 and 0.06 mg/kg of Se, respectively. Purified water supplied by an ultra-pure water supply system (Millipore Corporation, Darmstadt, Germany) or BSO-containing purified water were given for drinking ad libitum using water bottles.

All animal experimental procedures were approved by the Institutional Animal Care and Use Committee of the Toxicology Research Laboratories, Central Pharmaceutical Research Institute, JAPAN TOBACCO INC. (approval code 17084 (approved on 26 July 2017) and approval code 18091(approved on 24 July 2018)). This study was conducted in accordance with the Japanese Law for the Humane Treatment and Management of Animals (Law No. 105, as revised in 2013, issued in 1 October 1973).

### 4.3. Preparing Se/GSH-Depleted Rats

The study design was precisely described in Figure 1. Normal diet or Se-deficient diet were fed to rats for five weeks in total. After a four-week pre-feeding of each diet, purified water or 20 mmol/L BSO-containing purified water were given for a week to the control rats and Se/GSH-depleted rats, respectively. Necropsy was conducted on days 15 (Week 2), 29 (Week 4) and 36 (Week 5).

### 4.4. Toxicity Study of Flutamide Using Se/GSH-Depleted Rats

The dose level of flutamide was set at 150 mg/kg. Flutamide was suspended in an aqueous solution of 0.5% methylcellulose (Shin-etsu Chemical Co., Ltd., Tokyo, Japan, 5 mL/kg). The administration of flutamide was started 2 days after the initiation of treatment with BSO (dosing duration of flutamide: 5 days). Necropsy was conducted on day 36 (Week 5).

### 4.5. Sampling of Blood and Liver Samples

The abdomens of rats were opened under isoflurane anesthesia in non-fasted condition and blood samples were collected from the abdominal aorta. The blood samples to obtain serum were transferred into non-treated tubes and were left at room temperature for more than 30 min and then centrifuged at 1750 *g*, at room temperature for 30 min, to obtain the serum. The blood samples used to obtain plasma were transferred into tubes containing heparin as an anticoagulant and were centrifuged at 1750 *g* for 30 min at 4 °C to obtain plasma.

The livers were removed and liver samples were frozen with liquid nitrogen and stored at –80 °C until use. The remaining samples were preserved in neutral buffered formalin for histopathological examination.

### 4.6. Measurements of Serum Se Levels

The measurements of serum Se concentration were conducted by atomic absorption spectrophotometry by BML Inc. (Tokyo, Japan)

### 4.7. Measurements of Hepatic GSH and GSSG Levels

The frozen liver samples were thawed and a 5% 5-sulfosalicylic acid aqueous solution (Wako Pure Chemical) was added to the samples at a volume of 9 mL per 1 g wet tissue weight. The tissue samples were homogenized using a Tissuelyser System (QIAGEN, Hilden, Germany). The homogenates were centrifuged at 8000 *g* for 10 min at 4 °C and the supernatants were obtained for measurement of hepatic GSH and GSSG levels. Hepatic GSH and GSSG levels were measured using a GSH/GSSG quantification kit according to the manufacturer’s instructions (Dojindo laboratories, Kumamoto, Japan).

### 4.8. Measurements of Hepatic MDA Levels

The frozen liver samples were thawed and a RIPA buffer (Wako Pure Chemical) containing Halt^TM^ Protease Inhibitor Cocktail (Thermo Fisher Scientific, Waltham, MA, USA) was added to the samples at a volume of 1.5 mL per 25 mg wet tissue weight. The tissue samples were homogenized using a Tissuelyser System. The homogenates were centrifuged at 1600 *g* for 10 min at 4 °C and the supernatants were obtained for measurement of hepatic MDA levels. The hepatic MDA levels were measured using a TBARS Assay Kit according to the manufacturer’s instructions (Cayman Chemical, Ann Arbor, MI, USA).

### 4.9. Western Blotting

The frozen liver samples were thawed and a RIPA buffer containing Halt^TM^ Protease Inhibitor Cocktail was added to the samples at a volume of 4 mL per 1 g wet tissue weight. The tissue samples were homogenized using a Power Masher II and Bio Masher SP (Nippi, Tokyo, Japan). The homogenates were centrifuged at 13,000 *g* for 5 min at 4 °C and the supernatants were obtained for western blotting. The protein concentration of each sample was measured using a DC protein assay kit (Bio-Rad, Hercules, CA, USA). The samples of the supernatants mixed with EzApply (ATTO, Tokyo, Japan) were heated at 95 °C for 5 min and applied to 10% SDS polyacrylamide gels, and then the separated proteins were transferred to nitrocellulose membranes (Bio-Rad). After blocking for 30 min at room temperature with 3% skim milk in phosphate-buffered saline containing 0.05% Tween 20, the membranes were incubated overnight at 4 °C with rabbit antibodies against GPx-1, GPx-4 (1:1000, Abcam, Cambridge, UK) and β-actin (1:1000, Cell Signaling, Danvers, MA, USA) diluted by Can Get Signal I (TOYOBO Co. Ltd., Osaka, Japan). The membranes were subsequently incubated for 1 h at room temperature with peroxidase-conjugated anti-rabbit IgG antibody (1:5000, Cell Signaling) diluted by Can Get Signal II (TOYOBO Co. Ltd.). Immune complexes were visualized by an enhanced Immobilon™ Western (Merck Millipore, Bedford, MA, USA).

### 4.10. Measurements of Plasma Liver Function Parameters Levels

The measurements of plasma liver function parameters were conducted by an automated analyzer (TBA-120FR, TOSHIBA Corporation, Tokyo, Japan) using standard reagents for the clinical chemistry (Wako Pure Chemicals).

### 4.11. Histopathology

The livers were removed and preserved in neutral buffered formalin for histopathological examination. The liver slices were embedded in paraffin. Sectioning and hematoxylin-eosin staining was performed according to routine histological procedures.

### 4.12. Statistical Analysis

All numerical data are shown as mean ± or +/– standard deviation. The differences in the data were determined by Student’s *t*-test using GraphPad Prism 7 (7.04, GraphPad Software, La Jolla, CA, USA). The levels of significance were set at 5% and 1% (two-tailed).

## Figures and Tables

**Table 1:** The results of the profiling of Se/GSH-depleted rats.

Parameters	Group	Week 2	Week 4	Week 5
Serum Se level (μg/L)	Control	509.0 ± 34.8	550.0 ± 34.8	607.0 ± 69.7
	Se/GSH (−)	355.0 ± 4.6 **	396.0 ± 56.0 *	412.0 ± 79.7 *
Hepatic GSH level (μmol/g liver)	Control	2.90 ± 0.10	3.03 ± 0.06	3.53 ± 0.35
	Se/GSH (−)	5.37 ± 0.97 *	5.80 ± 0.89 **	1.43 ± 0.47 **
Hepatic GSSG level (μmol/g liver)	Control	0.95 ± 0.15	1.44 ± 0.56	1.47 ± 0.22
	Se/GSH (−)	0.16 ± 0.04 **	0.11 ± 0.03 *	0.61 ± 0.33 *
GSSG/GSH ratio	Control	0.33 ± 0.01	0.47 ± 0.17	0.41 ± 0.17
	Se/GSH (−)	0.03 ± 0.01 **	0.02 ± 0.002 *	0.41 ± 0.10
Hepatic MDA level (×10^−2^ μmol/g liver)	Control	13.7 ± 0.3	12.6 ± 0.4	12.2 ± 0.5
	Se/GSH (−)	13.4 ± 0.1	14.4 ± 0.4 **	14.3 ± 0.4 **
Plasma AST level (U/L)	Control	75.7 ± 1.5	57.3 ± 6.5	59.0 ± 7.5
	Se/GSH (−)	65.7 ± 8.6	60.0 ± 57.3	55.7 ± 1.5
Plasma ALT level (U/L)	Control	25.7 ± 4.0	18.3 ± 2.5	22.3 ± 7.4
	Se/GSH (−)	21.0 ± 3.0	21.0 ± 1.7	17.3 ± 3.2
Plasma T-BIL level (mg/dL)	Control	0.047 ± 0.006	0.053 ± 0.021	0.053 ± 0.025
	Se/GSH (−)	0.043 ± 0.006	0.050 ± 0.010	0.053 ± 0.021
Plasma GLDH level (U/L)	Control	18.3 ± 1.2	16.7 ± 2.3	15.7 ± 2.9
	Se/GSH (−)	14.7 ± 2.9	17.0 ± 4.4	15.0 ± 2.6
Plasma SDH level (U/L)	Control	1.17 ± 0.76	2.17 ± 1.44	4.33 ± 1.53
	Se/GSH (−)	1.67 ± 1.15	2.17 ± 2.47	4.00 ± 2.00

Mean ± S.D. are presented with 5 determinations. * *p* < 0.05, ** *p* < 0.01 (Student’s *t*-test). Se: Selenium, GSH: Glutathione, GSSG: Glutathione disulfide, MDA: Malondialdehyde, AST: Aspartate aminotransferase, ALT: Alanine aminotransferase, T-BIL: Total-bilirubin, GLDH: Glutamate dehydrogenase, SDH: Sorbitol dehydrogenase, Se/GSH (−): Se/GSH-depleted group.

**Table 2:** The histopathological findings of the liver in the Se/GSH (+) and (−) rats treated with flutamide.

Dose Level of Flutamide (mg/kg/day)	Se/GSH (+)		Se/GSH (−)	
	0	150	0	150
Hypertrophy, hepatocyte, centrilobular	−: 5	±: 3	−: 5	±: 2
		+: 2		+: 3
Infiltration, inflammatory cell, periportal	−: 5	−: 5	−: 5	−: 4
				±: 1
Single cell necrosis, hepatocyte, centrilobular	−: 5	−: 5	−: 5	−: 4
				±: 1
Necrosis, hepatocyte, focal, subcapsular	−: 5	−: 5	−: 5	−: 5
				+: 1

−: Finding absent, ±: very slight, +: slight. Se/GSH (+): normal rats, Se/GSH (−): Se/GSH-depleted rats.
